# Vitamin D deficiency induces Th2 skewing and eosinophilia in neonatal allergic airways disease

**DOI:** 10.1111/all.12465

**Published:** 2014-08-04

**Authors:** J E Vasiliou, S Lui, S A Walker, V Chohan, E Xystrakis, A Bush, C M Hawrylowicz, S Saglani, C M Lloyd

**Affiliations:** 1 Leukocyte Biology Section, National Heart and Lung Institute, Imperial College LondonLondon, UK; 2 MRC & Asthma UK Centre for Allergic Mechanisms of Asthma, King's College London, Guy's HospitalLondon, UK; 3 Respiratory Paediatrics, Royal Brompton Hospital, and National Heart & Lung Institute, Imperial College LondonLondon, UK

**Keywords:** animal models, asthma, eosinophils, lymphocytes, paediatric

## Abstract

**Background:**

Associations between vitamin D status and childhood asthma are increasingly reported, but direct causation and mechanisms underlying an effect remain unknown. We investigated the effect of early-life vitamin D deficiency on the development of murine neonatal allergic airways disease (AAD).

**Methods:**

*In utero* and early-life vitamin D deficiency was achieved using a vitamin D-deficient diet for female mice during the third trimester of pregnancy and lactation. Offspring were weaned onto a vitamin D-deficient or vitamin D-replete diet, and exposure to intranasal house dust mite (HDM) or saline was commenced from day 3 of life for up to 6 weeks, when airway hyper-responsiveness (AHR), airway inflammation and remodelling were assessed.

**Results:**

Neonatal mice that had *in utero* and early-life vitamin D deficiency had significantly increased pulmonary CD3^+^CD4^+^T1ST2^+^ cells and reduced CD4^+^IL-10^+^ cells. This effect was enhanced following HDM exposure. AHR in HDM-exposed mice was unaffected by vitamin D status. Introduction of vitamin D into the diet at weaning resulted in a significant reduction in serum IgE levels, reduced pulmonary eosinophilia and peri-bronchiolar collagen deposition.

**Conclusion:**

Peri-natal vitamin D deficiency alone has immunomodulatory effects including Th2 skewing and reduced IL-10-secreting T regulatory cells, exaggerated with additional allergen exposure. Vitamin D deficiency in early life does not affect AHR, but contributes to disease severity with worse eosinophilic inflammation and airway remodelling. Importantly, supplementation with vitamin D improves both of these pathological abnormalities.

Asthma is a chronic airway inflammatory disease which commonly begins in early life. Growing evidence suggests that both genetic and environmental factors contribute to the development and severity of asthma, and exposures during the peri-natal period are highly influential (1). Vitamin D is primarily derived from skin synthesis in response to sunlight in humans, and deficiency has been implicated in many diseases including pulmonary diseases involving both infection and allergy (2). An association between vitamin D receptor (VDR) gene polymorphisms and asthma susceptibility is reported (3, 4); however, the relationship between serum vitamin D levels and asthma remains controversial (5). An inverse association between serum vitamin D and total IgE, lung function and asthma exacerbations in physician diagnosed asthmatic children have been described (6–8). Low serum vitamin D in childhood is thought to be predictive of asthma in adolescent males (9). Conversely, increased asthma was reported in adulthood after a period of vitamin D supplementation during the first year of life (10). A recent unselected cohort of over 2000 children observed no association between serum vitamin D and asthma or lung function; however, higher serum vitamin D was associated with a greater risk of wheeze (11). Serum vitamin D levels have been associated with disease severity in children and were reduced in severe compared to moderate asthmatics (12). Additionally, lower serum vitamin D was associated with poorer lung function and increased sensitization to aeroallergens (12, 13). Maternal vitamin D status during pregnancy may also determine risk of asthma development rather than the child's own vitamin D levels. Reduced maternal vitamin D during pregnancy has been associated with an increase in early wheeze, but this association does not extend to asthma at school age (14–16). Cord blood vitamin D levels inversely correlated with wheeze (17), while a U-shaped relationship with total and antigen-specific IgE has been observed in a population of children in Arizona (18). Conversely, maternal vitamin D status has been reported to have no associations with asthma or wheeze (19, 20). The variation in reported findings, and reliance on associations, rather than proof of causation, has prevented any firm conclusions about the impact of childhood or maternal vitamin D status on asthma development in children.

The role of vitamin D as an immunomodulator is better defined, with vitamin D skewing the immune system towards regulation. Treatment of murine CD4^+^ T cells with vitamin D promotes IL-10 gene expression (21) and protein (22), while treatment of human CD4^+^ T cells with vitamin D also results in IL-10 secretion (23). Multiple studies have reported a positive correlation between serum vitamin D and FoxP3^+^ regulatory T-cell numbers in both the periphery and airways of adults (24) and children (25, 26).

As diet is the only source of vitamin D for rodents (without a contribution from ultraviolet light), we used a vitamin D-deficient diet to alter vitamin D status. Pregnant females were fed a vitamin D-deficient diet starting from day-16 gestation (last trimester) to focus on immune-mediated mechanisms of disease development in offspring and to limit any effects of *in utero* vitamin D deficiency on foetal lung growth and development (27). To determine whether vitamin D deficiency exacerbates allergic airways disease (AAD), and the mechanisms underlying this, we used a neonatal model of inhaled house dust mite (HDM) exposure (28). We hypothesized that pups experiencing *in utero* and early-life vitamin D deficiency would develop more severe HDM-induced airway hyper-responsiveness (AHR), eosinophilic inflammation and remodelling due to a reduction in regulatory T cells in the lung.

## Methods

### Animals and allergen challenge

**Figure 1:**
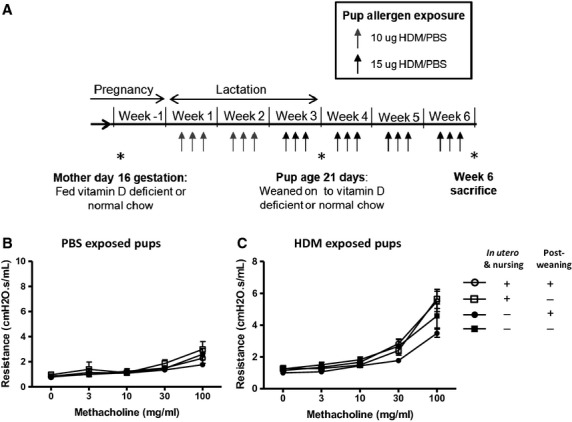
Early-life vitamin D insufficiency has no effect on airway resistance in pups at 6 weeks of age. Schematic representation of experimental protocol (A). Pregnant female Balb/c mice were fed a vitamin D-deficient diet from day-16 gestation or remained on a normal chow diet. Pups were challenged with house dust mite (HDM) or PBS for 6 weeks. Pups were weaned at 3 weeks of age and either fed a vitamin D-deficient diet or a normal chow diet. At 6 weeks of age, lung function was measured by the forced oscillation technique 4 h after last challenge of PBS (B) or HDM (C). + represents vitamin D intake and - represents a lack of vitamin D. Data are expressed as mean and SEM, *n* = 4–7.

Balb/c mice (Harlan, Bicester) were maintained by in-house breeding. Female Balb/c mice were mated and, from day-16 gestation, were either fed a vitamin D-deficient chow (Special Diets Services, UK) or remained on a regular diet throughout pregnancy and lactation. Vitamin D deficiency is achieved within 4 weeks of feeding a vitamin D-deficient diet (29). Resulting pups of similar weights were exposed to intranasal administration of either 10 μg (in 10 μl of saline (PBS) HDM extract (Greer, Lenior, NC, USA)) or 10 μl PBS, three times a week for the first 2 weeks starting from day 3 of life. HDM dose was increased to 15 μg from week 3 onwards (28) and continued for a total of 6 weeks (Fig. 1A). All experiments were conducted in accordance with United Kingdom Home Office regulations and approved by the Imperial College London Animal Welfare and Ethical Review Body (AWERB). Animals had free access to food and water and were kept under a 12-h light and dark cycle.

### Lung function

Airway hyper-responsiveness was measured 4 h after the final HDM or PBS challenge in response to increasing doses of methacholine (3–100 mg/ml; Sigma-Aldrich, Gillingham, UK) using the forced oscillation technique on a flexivent system (Scireq, Montreal, QC, Canada) as described previously (28).

### Cell recovery

Bronchoalveolar lavage (BAL) was performed using three aliquots of saline via a tracheal cannula (28). BAL fluid was centrifuged, supernatants removed, and cell pellets resuspended in 0.5 ml complete media (RPMI/10% FCS/2 mM l-glutamine/100 U/ml penicillin/streptomycin). One lobe of lung tissue was mechanically chopped and incubated at 37°C for 1 h in complete media containing 0.15 mg/ml collagenase (Type D; Roche Diagnostics, West Sussex, UK) and 25 μg/ml DNAse (Type 1; Roche Diagnostics). Recovered cells were filtered through a 70-um nylon sieve, washed twice and resuspended in 1 ml complete media.

### Flow cytometry

Lung cells were stained in PBS/1% FCS/0.01% sodium azide. Cells were incubated with rabbit serum (Sigma-Aldrich) for 15 min before staining with FITC-T1ST2 (Morwell Diagnostics, Zurich, Switzerland), PE-CD8 (BD Biosciences, Oxford, UK), PerCP-Cy5.5-CD4 (eBioscience, Hatfield, UK) or APC-CD3e (BD Biosciences) or relevant isotype controls for 20 min at 4°C. Cells were washed twice and fixed in fixation buffer (eBioscience). For intracellular cytokine staining, cells were stimulated with PMA (Sigma-Aldrich)/Ionomycin (Emdchemicals Inc, San Diego, CA, USA) in the presence of Brefeldin A (Sigma-Aldrich) for 3 h before extracellular staining. Thereafter, cells were permeabilized using permeabilization buffer (eBioscience) and stained with PE-IL-10 (BD Pharmingen), or appropriate isotype control. Analysis was performed using a FACS ARIA (BD Biosciences) and FlowJo software (v7.6.5; Tree Star, Ashland, OR, USA).

### Cytokines and immunoglobulins

Lung tissue was homogenized at 50 mg/ml in HBSS (Gibco, Life Technologies, Paisley, UK) containing protease inhibitor tablets (Roche Diagnostics) and centrifuged at 800 ***g*** for 20 min, and supernatants were collected. Cytokines were analysed in lung homogenate supernatants and antibodies in serum. Paired antibodies for murine IgE, interleukin (IL)-4, IL-5, IFN-γ (BD Biosciences), IL-33, IL-10 (R&D Systems, Abingdon, UK), and IL-13 and IL-25 (ebioscience) were used in standardized sandwich ELISAs according to the manufacturer's protocol. Serum HDM-specific IgE was analysed by a previously published method (28).

### Goblet cell hyperplasia

Goblet cells were scored on sections stained with periodic acid–Schiff, using a semiquantitative scoring system as previously described (28).

### Lung collagen quantification

Total lung collagen was measured in lung homogenates using the sircol assay (Biocolor Life Science Assays, County Antrim, UK) as per manufacturer's instructions. Peri-bronchiolar collagen was quantified on sections stained with Sirius red and a polarizing lens, with computer-aided image analysis (Leica Microsystems, Milton Keynes, UK) as previously described (30).

### Statistical analysis

Data were analysed using GraphPad Prism 5 software (GraphPad Software, San Diego, CA, USA). Nonparametric tests (Mann–Whitney U) were used to detect differences between groups and statistical significance accepted when *P* < 0.05, *n* = 4–7 animals per group with experiments carried out twice. Where data have been represented as box and whisker plots, the horizontal bar represents the median value, the whiskers represent the maximum and minimum value, and the box represents the interquartile range.

## Results

### Vitamin D insufficiency skews towards a pulmonary Th2 phenotype and reduces IL-10^+^ T regulatory cells in pregnant females

Pregnant female Balb/c mice received a vitamin D-deficient diet from day-16 gestation and throughout lactation for a total of 4 weeks to induce vitamin D insufficiency (Fig. 1A) (29). Vitamin D insufficiency did not affect total lung or bronchoalveolar lavage (BAL) cells (Fig. S1A), but did result in a significant increase in pulmonary CD3^+^CD4^+^T1ST2^+^ Th2 cells and a reduction in CD4^+^IL-10^+^ T regulatory cells (Fig. S1B).

### Vitamin D insufficiency in early life does not alter AHR in neonatal mice exposed to HDM

Early-life vitamin D insufficiency did not affect airway resistance in PBS- or HDM-exposed pups at 6 weeks of age (Fig. 1A–C). Lung compliance was also unaffected by vitamin D status (data not shown).

### Vitamin D supplementation after weaning reduces pulmonary eosinophilia

**Figure 2:**
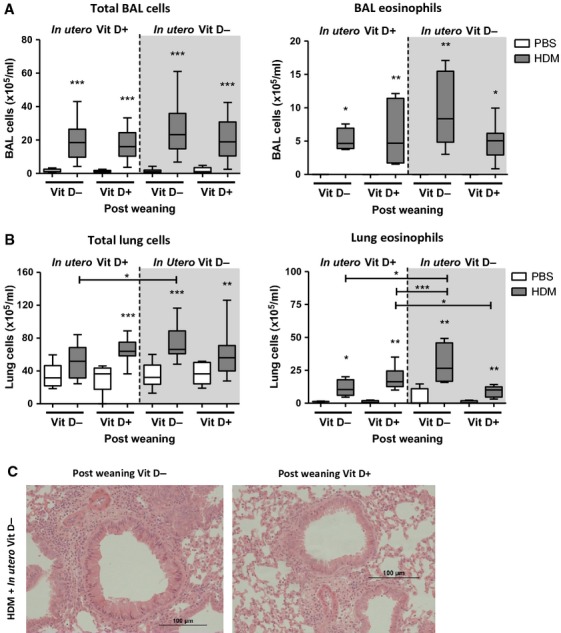
Introduction of dietary vitamin D after *in utero* and early-life insufficiency can reduce eosinophilic lung inflammation. Pregnant female Balb/c mice were either fed a vitamin D-deficient diet from day-16 gestation or remained on a normal chow diet. Pups were exposed to house dust mite (HDM) or PBS and weaned on to either a vitamin D-deficient diet or a normal chow at 3 weeks of age. At 6 weeks of age, both total and eosinophil numbers in the BAL (A) and lung (B) were counted. + represents vitamin D intake; - represents a lack of vitamin D. Lung sections stained with haematoxylin and eosin from HDM-exposed pups with *in utero* vitamin D insufficiency (C). Postweaning vitamin D insufficiency (left) and postweaning vitamin D sufficiency (right) are shown. Data are expressed as box and whisker plots; horizontal bar represents median, *n* = 9–16 combined from 2 experiments. *P < 0.05, **P < 0.01, ***P < 0.001 by Mann–Whitney U-test. Significance refers to HDM and associated PBS control where no line is indicated.

Inflammation was determined in mice following 6 weeks of allergen exposure and ingestion of either a normal or vitamin D-deficient diet. Lung eosinophilia was highest in pups after *in utero* and early-life insufficiency, and this was significantly reduced when pups were supplemented with vitamin D at weaning (Fig. 2B). This was true for both total and percentage of eosinophils in the lung (Figs 2B and S2). Eosinophils in the BAL (Fig. 2A) and total numbers of inflammatory cells in both the BAL and lung were unaffected by vitamin D status (Fig. 2A,B).

### Vitamin D insufficiency *in utero* and in early life promotes pulmonary Th2 skewing and reduced IL-10^+^ T regulatory cells

**Figure 3:**
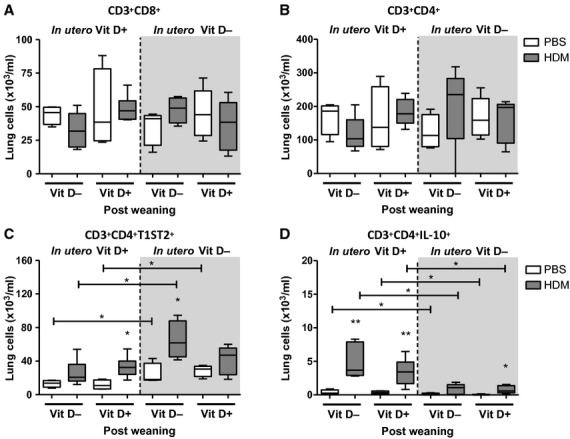
*In utero* and early-life vitamin D insufficiency skews towards a Th2 environment and reduces T regulatory cells in the lungs of pups. Pregnant female Balb/c mice were fed a vitamin D-deficient diet from day-16 gestation or remained on a normal chow diet. Pups were challenged with house dust mite (HDM) or PBS for 6 weeks. Pups were weaned at 3 weeks of age and either fed a vitamin D-deficient diet or a normal chow. Lung cells were harvested 4 h after last challenge and analysed by flow cytometry. T-cell subsets were defined as CD3^+^CD8^+^ (A), CD3^+^CD4^+^ (B), CD3^+^CD4^+^T1ST2^+^ (C) and CD3^+^CD4^+^IL-10^+^ (D). + represents vitamin D intake; - represents a lack of vitamin D. Data are expressed as box and whisker plots; horizontal bar represents median, *n* = 4–6. *P < 0.05, **P < 0.01 by Mann–Whitney U-test. Significance refers to HDM and associated PBS control where no line is indicated.

To determine the role of vitamin D in T-cell differentiation, we analysed Th2 cells and IL-10^+^ T regulatory cells in the lungs of pups. Total CD8^+^ T cells and CD4^+^ T cells remained unchanged across all groups, regardless of dietary status (Fig. 3A,B). However, both the total cell count and percentage of CD3^+^CD4^+^T1ST2^+^ Th2 cells were significantly increased in pups exposed to *in utero* and early-life vitamin D insufficiency (Figs 3C and S2). Conversely, pulmonary CD3^+^CD4^+^IL-10^+^ T regulatory cells were significantly reduced in pups exposed to *in utero* and early-life vitamin D insufficiency (Fig. 3D). Both the increase in Th2 cells and the reduction in T regulatory cells occurred regardless of allergen exposure. A vitamin D-replete diet from 3 weeks of age for a further 3 weeks did not alter this phenotype.

### Supplementation with vitamin D after *in utero* and early-life insufficiency reduces serum IgE in pups at 6 weeks of age, but not Th2 cytokines

**Figure 4:**
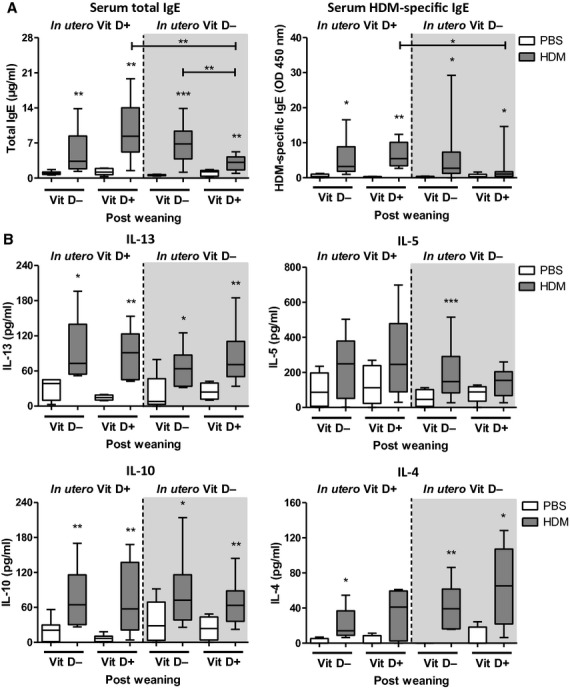
Introduction of dietary vitamin D after *in utero* and early-life insufficiency can reduce IgE in pups at 6 weeks of age. Pregnant female Balb/c mice were fed a vitamin D-deficient diet from day-16 gestation or remained on a normal chow diet. Pups were challenged with house dust mite (HDM) or PBS for 6 weeks. Pups were weaned at 3 weeks of age and either fed a vitamin D-deficient diet or a normal chow. Total IgE and HDM-specific IgE (A) antibodies were measured in the serum of pups by ELISA. The cytokines IL-13, IL-5, IL-10 and IL-4 (B) were measured in the lung of pups by ELISA. + represents vitamin D intake; - represents a lack of vitamin D. Data are expressed as box and whisker plots; horizontal bar represents median, *n* = 9–16 combined from 2 experiments. *P < 0.05, **P < 0.01, ***P < 0.001 by Mann–Whitney U-test. Significance refers to HDM and associated PBS control where no line is indicated.

We measured serum total and HDM-specific IgE to assess whether early-life vitamin D insufficiency affected sensitization to allergen (Fig. 4A). All pups exposed to HDM had increased levels of total and HDM-specific IgE compared to PBS controls. A significant reduction in total IgE was evident if vitamin D was introduced into the diet at weaning.

The skew in numbers of Th2 cells in pups exposed to *in utero* and early-life vitamin D insufficiency was not evident in the level of pulmonary Th2 cytokines IL-13, IL-5 or IL-4 (Fig. 4B). Additionally, pulmonary IL-10 remained the same in all HDM challenged pups and was not influenced by vitamin D status even though numbers of CD4^+^IL-10^+^ cells were reduced with vitamin D insufficiency (Figs 3D and 4B). IFN-γ, eotaxin-2, IL-25 and IL-33 (Fig. S2) were not influenced by vitamin D status. These data suggest that although lung cytokine levels may not change, the cellular source of cytokines and intimate contact between infiltrating inflammatory cells and resident stromal cells may be more important in determining an effector response.

### Vitamin D supplementation reduces airway collagen in HDM-exposed pups

**Figure 5:**
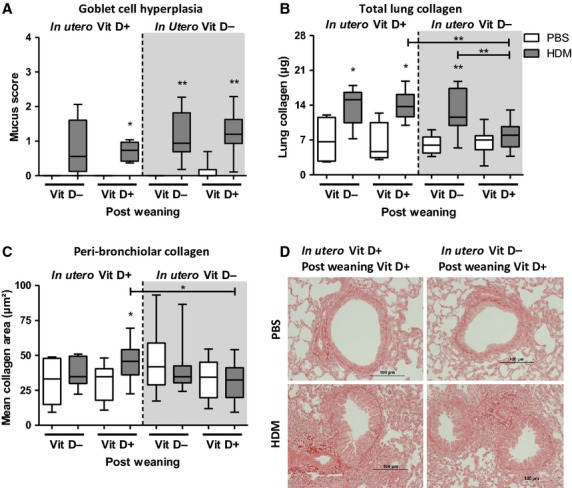
Introduction of dietary vitamin D after an *in utero* and early-life insufficiency reduces house dust mite (HDM)-induced lung collagen. Pregnant female Balb/c mice were fed a vitamin D-deficient diet from day-16 gestation or remained on a normal chow diet. Pups were challenged with HDM or PBS for 6 weeks. Pups were weaned at 3 weeks of age and either fed a vitamin D-deficient diet or a normal chow. At 6 weeks of age, lung sections were collected from pups 4 h after last challenge. Periodic acid–Schiff (PAS) staining was used to determine goblet cell hyperplasia in the lungs using a semiquantitative scoring system (A). Collagen deposition in the lungs of pups was measured using the Sircol assay for total lung collagen (B) and by image quantification to measure peri-bronchiolar-associated collagen (C). Lung sections stained with Sirius red (D); + represents vitamin D intake; - represents a lack of vitamin D. Data are expressed as box and whisker plots; horizontal bar represents median, *n* = 9–16 combined from 2 experiments. *P < 0.05, **P < 0.01 by Mann–Whitney U-test. Significance refers to HDM and associated PBS control where no line is indicated.

Goblet cell hyperplasia was evident in HDM-exposed pups; however, vitamin D status did not alter this (Fig. 5A). *In utero* and early-life vitamin D insufficiency did not alter lung collagen content in HDM-exposed pups. However, pups that were weaned onto a vitamin D-replete diet at 3 weeks of age after *in utero* and early-life insufficiency had reduced levels of lung collagen following 6 weeks of allergen exposure (Fig. 5B). The same reduction was still evident when peri-bronchiolar collagen was quantified (Fig. 5C,D), suggesting vitamin D supplementation may protect from the development of airway remodelling.

## Discussion

We have shown that adult female mice given a vitamin D-deficient diet for 4 weeks (from day-16 gestation until 3 weeks postpartum) developed a Th2-skewed pulmonary immune phenotype, concomitant with reduced CD4^+^IL-10^+^ regulatory cells. Moreover, offspring from vitamin D insufficient females exposed to HDM and fed a vitamin D-deficient diet had increased pulmonary eosinophilia, Th2 cells and reduced CD4^+^IL-10^+^ regulatory cells. However, this skewed immune phenotype did not result in worse AHR. Introduction of vitamin D after *in utero* and early-life deficiency lowered disease severity in HDM-exposed neonatal mice by reducing eosinophilic inflammation, airway remodelling and serum IgE levels.

Dietary vitamin D deficiency in an adult model of ovalbumin-induced AAD demonstrated worse AHR, eosinophilia and reduced IL-10 levels (31). However, the diet was used for 13 weeks prior to allergen exposure. We have shown effects on immune phenotype after only 4 weeks of a vitamin D-deficient diet in females. Ours is the first study to investigate the impact of maternal vitamin D status on early-life allergic inflammation. A previous study has investigated the effect of *in utero* and early-life vitamin D deficiency on AAD, but allergen challenge was commenced in adult mice, rather than during the neonatal period (32). Despite establishment of vitamin D-replete and vitamin D-deficient mice through 5 weeks of dietary manipulation of parent mice before breeding, and from birth of offspring until adulthood, ovalbumin exposure did not impact lung function in offspring. This concurred with our findings showing allergen exposure in neonatal mice with *in utero* and postnatal vitamin D deficiency did not affect lung function. However, findings are in contrast to adult deficient mice where AHR was increased (31). Isolated vitamin D deficiency throughout gestation has been shown to cause deficits in lung function in 2-week-old mouse offspring (27). However, our data show that there is no additional effect on lung function with allergen exposure. This highlights the importance of age and vitamin D status on lung function in adults compared to *in utero* and early-life deficiency. It is consistent with findings from epidemiological studies which have failed to show reliable effects of vitamin D deficiency or insufficiency on lung function in children with asthma (6–8, 11) although associations are reported in adults (33). Overall, given our findings in a neonatal mouse model, and previous reports of vitamin D deficiency *in utero* through adulthood not resulting in significantly worse AHR, it seems likely that in the context of asthma in early life, vitamin D deficiency modifies immunological responses to allergen by the induction of an exaggerated Th2 skewed response and eosinophilia.

Studies in asthmatic children have shown associations between low vitamin D status and increased use of inhaled steroids (34), suggesting the immunomodulatory effects of vitamin D may impact on efficacy of steroids. Data from adult studies show an association between vitamin D status and steroid response (23). Children with severe asthma on high-dose steroid therapy have worse clinical features with lower serum vitamin D levels (12). Previous work has demonstrated that vitamin D3 supplementation potentiates the benefits of allergen immunotherapy in allergic airways disease (35). An important finding from our data is the partial protection from disease severity seen in pups that were born to mothers on a vitamin D-deficient diet, but were weaned onto a vitamin D-replete diet. They had deficiency *in utero* and in early life, but then had supplementation from 3 to 6 weeks of age (when weaned). This resulted in lower eosinophilia, less airway remodelling and lower serum IgE levels compared to pups that remained on a vitamin D-deficient diet after weaning. Our data suggest vitamin D is playing a widespread role in immunomodulation, and further suggests that supplementation may be beneficial and may reduce disease severity.

An interesting point to note is that after introduction of a vitamin D-replete diet postweaning, pulmonary eosinophilic inflammation was even lower than in mice that were always vitamin D sufficient (Fig. 2A). Similarly, serum IgE levels were lowest in mice that were supplemented with vitamin D at weaning, even lower than those that were always vitamin D sufficient (Fig. 4A). The same effect was seen in a previous study that has investigated the effects of vitamin D supplementation on allergic airways disease in an ovalbumin model using adult mice. That study by Agrawal and colleagues has shown that the improvement in inflammation is significantly better in mice that are supplemented with vitamin D compared to controls that were vitamin D sufficient (31). A mechanism for this finding was not reported, but in agreement with our findings, vitamin D supplementation in that model also only showed an improvement in allergic inflammation, but not a complete resolution down to levels in nonallergic mice, and thus, the authors conclude vitamin D supplementation is likely an important adjunct in the treatment of asthma, rather than a candidate that will achieve a cure. Similar findings have not been reported from trials of vitamin D supplementation in humans as they only include patients that have deficient or insufficient levels prior to an intervention with vitamin D or placebo. A vitamin D-sufficient positive control group is therefore not reported. Models of autoimmune disease have either supplemented vitamin D-sufficient animals and shown benefit or only compared responses in vitamin D-deficient animals, without a sufficient positive control group.

An important pathological feature of asthma, for which therapies are currently unavailable, is airway remodelling. We have shown a reduction in both total lung collagen and peri-bronchiolar collagen in mice that were exposed to HDM and had vitamin D supplementation after weaning. A reduction in airway remodelling has been shown in adult ovalbumin-induced AAD with additional vitamin D therapy (36). It is interesting that in our study, dietary vitamin D *in utero* was associated with the highest numbers of IL-10-secreting Tregs. Supplementation of dietary vitamin D postweaning resulted in significantly higher IL-10^+^ Tregs in HDM-exposed pups. However, numbers of CD4^+^IL-10^+^ cells were not significantly higher in the supplemented mice compared to those that remained vitamin D deficient. This may be explained by the impact on local, tissue-specific T cells in a disease situation, rather than circulating T cells. A pilot study of high-dose vitamin D supplementation in adults (*n* = 4) has shown an increase in circulating IL-10-producing peripheral blood mononuclear cells (PBMCs) (37); however, these were otherwise healthy subjects and only circulating cells were assessed. Daily vitamin D3 supplementation for 3 months in patients with multiple sclerosis did result in an increased proportion of CD4^+^IL-10^+^ Tregs, but these were also circulating cells and not site specific (38). An *in vivo* study investigating the mechanism by which vitamin D3 prevents experimental autoimmune encephalitis has shown that myelin-reactive T helper cells are successfully generated in the presence of vitamin D3, secrete pro-inflammatory cytokines and do not preferentially differentiate into suppressor T cells. However, vitamin D3 prevents entry of pro-inflammatory T helper cells into the central nervous system (CNS), and instead, they are maintained in the periphery. This halting of T helper cell migration to the CNS is central to the beneficial effects of vitamin D3 (39). In addition, an adult murine model of vitamin D supplementation in ovalbumin-induced allergic airways disease demonstrated an increase in circulating T regulatory cells after vitamin D supplementation, but levels of pulmonary Tregs were not reported (31). We have only assessed alterations in pulmonary IL-10-producing T cells as this is the site of the allergen exposure, but did not see an increase in these. It may be that only alterations in circulating T regulatory cells are apparent following supplementation with vitamin D.

We have previously shown that transfer of T regulatory cells prevented development of airway remodelling in an adult mouse model of AAD (40). A promising role of vitamin D supplementation in children with asthma is the potential of minimizing the development of a very early feature of airway remodelling, increased thickness of the reticular basement membrane (41, 42), which would incorporate a novel therapeutic target in asthma.

In summary, we report for the first time that *in utero* and postnatal vitamin D deficiency, with concomitant inhaled allergen exposure in the neonatal period, results in increased airway eosinophilia and Th2 cells and reduced CD4^+^IL-10^+^ T regulatory cells. However, AHR is unaffected by vitamin D deficiency in neonatal AAD. Our data suggest vitamin D deficiency *in utero* and early life contributes to worse eosinophilic inflammation and that vitamin D supplementation after weaning reduces the development of both pulmonary eosinophilia and remodelling. Therefore, although it does not seem likely that vitamin D deficiency *per se* causes asthma in early life, it is likely that vitamin D deficiency contributes to increased disease severity in children with asthma and that supplementation may reduce severity.

## Supporting Information

Figure S1 Vitamin D insufficiency skews towards a Th2 response, and reduces IL-10 producing T regulatory cells in the lung.

Figure S2 Vitamin D plays an immunomodulatory role in the lung of pups.

Figure S3 Cytokine levels in the lung are not altered by early-life vitamin D insufficiency.

Figure S4 *In utero* and early-life vitamin D insufficiency does not alter lung Th17 cells.
