# Supplementary figures and images for: Vitamin D deficiency induces Th2 skewing and eosinophilia in neonatal allergic airways disease
Source: Allergy. 2014 Aug 4;69(10):1380–9. doi: 10.1111/all.12465 (PMC4329404; doi:10.1111/all.12465)

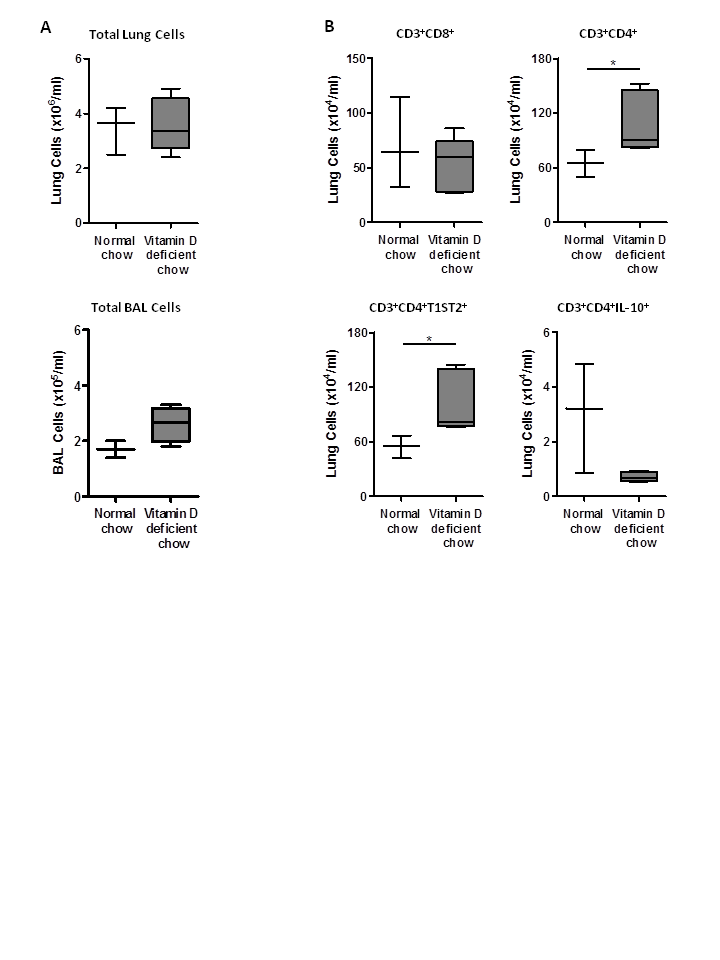

Supplement: Figure S1 — Vitamin D insufficiency skews towards a Th2 response, and reduces IL-10 producing T regulatory cells in the lung. [file all0069-1380-sd1.gif]

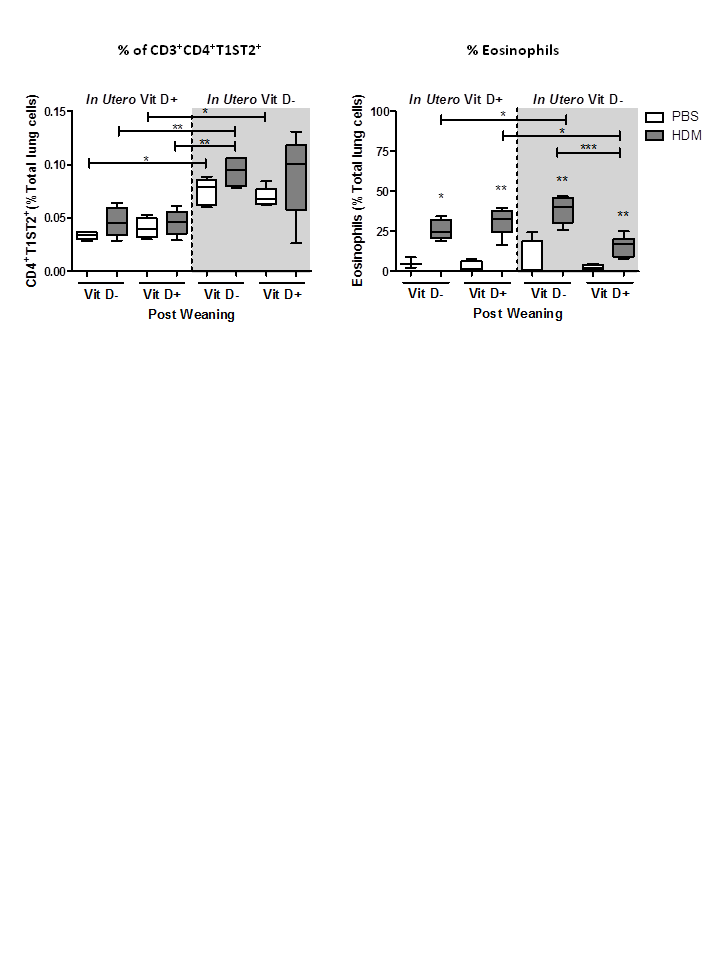

Supplement: Figure S2 — Vitamin D plays an immunomodulatory role in the lung of pups. [file all0069-1380-sd2.gif]

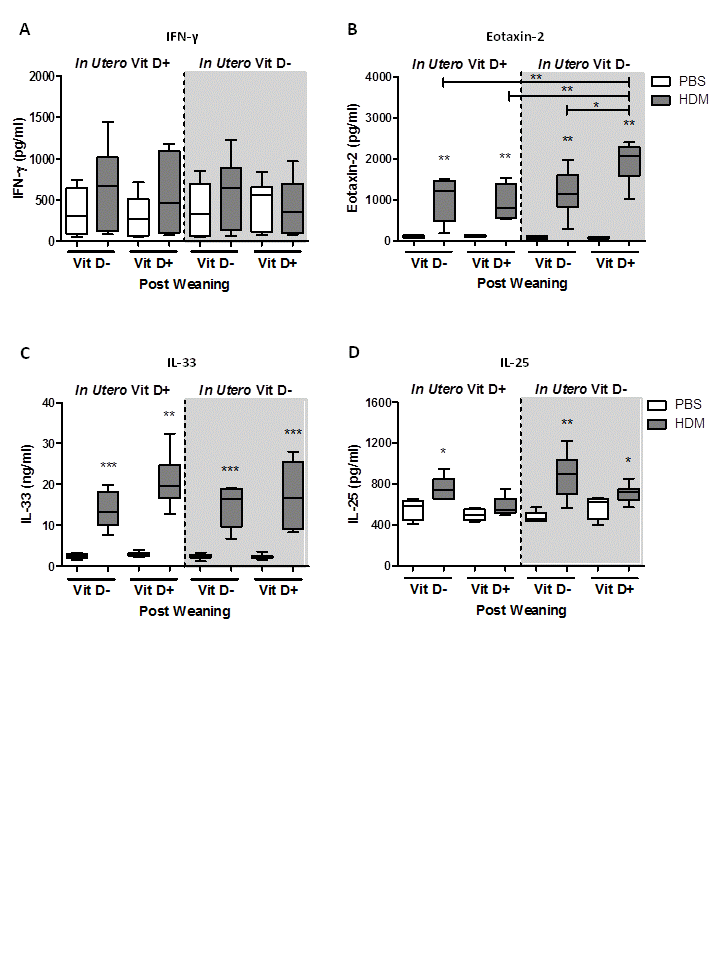

Supplement: Figure S3 — Cytokine levels in the lung are not altered by early-life vitamin D insufficiency. [file all0069-1380-sd3.gif]

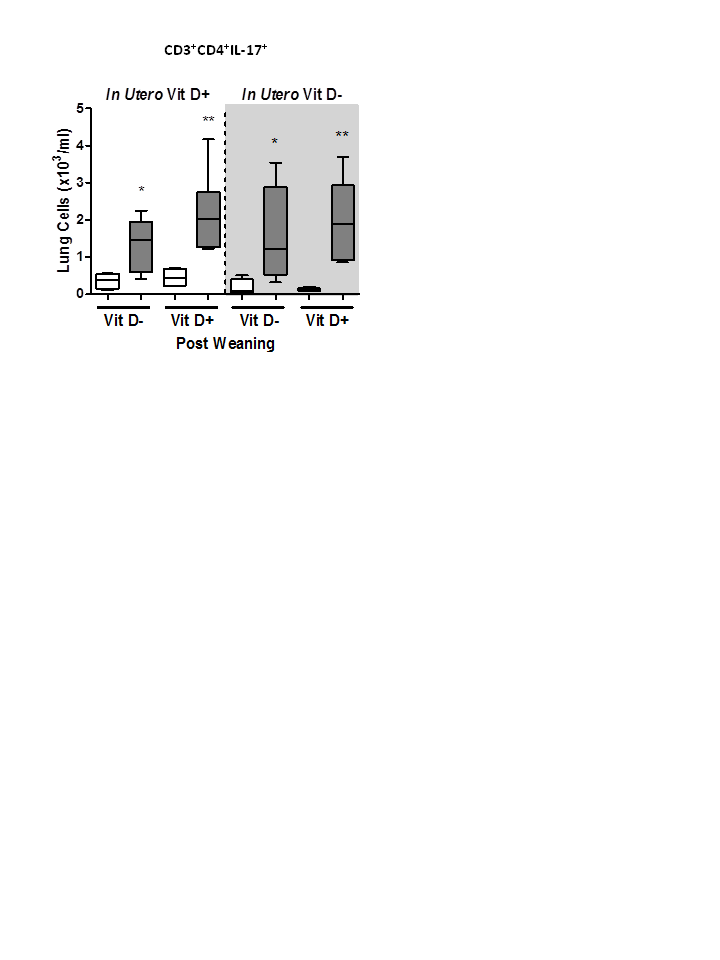

Supplement: Figure S4 — In utero and early-life vitamin D insufficiency does not alter lung Th17 cells. [file all0069-1380-sd4.gif]
